# Effects and possible mechanisms of *Alpinia officinarum* ethanol extract on indomethacin-induced gastric injury in rats

**DOI:** 10.1080/13880209.2018.1450426

**Published:** 2018-05-21

**Authors:** Jingwen Gong, Zhong Zhang, Xuguang Zhang, Feng Chen, Yinfeng Tan, Hailong Li, Jie Jiang, Junqing Zhang

**Affiliations:** Hainan Provincial Key Laboratory of R&D of Tropical Herbs, Hainan Medical University, Haikou, China

**Keywords:** Nonsteroidal anti-inflammatory drugs, cyclooxygenase, nitric oxide

## Abstract

**Context:***Alpinia officinarum* Hance (Zingiberoside) has a long history in treating gastrointestinal diseases, but its mechanisms of action are not yet known.

**Objective:** To investigate the effects and underlying mechanisms of the ethanol extract of *A. officinarum* rhizomes in an indomethacin-induced gastric injury rat model.

**Material and methods:** Indomethacin (0.3 g/kg) was orally administered to Sprague-Dawley rats to induce gastric damage; after 7 h, the rats were treated with 0.03, 0.09, or 0.18 g/kg of the plant extract, galangin (0.2 g/kg), or bismuth potassium citrate (0.08 g/kg), once a day for 6 days. Rats in the control group received an equivalent volume of vehicle solution for 6 days. Gastric damage was evaluated by gross ulcer and histological indexes. Cyclooxygenase and non-cyclooxygenase pathway proteins were quantified by western blotting and ELISA.

**Results:***Alpinia officinarum* extract ameliorated gastric injury in a dose-dependent manner, and 0.18 g/kg dose exhibited the best performance by reducing the gross ulcer (from 20.23 ± 1.38 to 1.66 ± 0.37) and histological (from 4.67 ± 1.03 to 0.33 ± 0.51) indexes, decreasing serum TNF-α level (14.17%), increasing serum VEGF level (1.58 times), increasing cyclooxygenase-1 level (1.25 times, *p* <  0.001) in the gastric mucosa, and reversing indomethacin-induced changes in the expression of non-cyclooxygenase pathway proteins (*p* <  0.05). Galangin was less effective as an antiulcer agent than the whole extract, indicating that other components also contributed to the protective effect.

**Conclusions:***Alpinia officinarum* extract and galangin exert antiulcer effects through cyclooxygenase and non-cyclooxygenase pathways validating use of galangin as a treatment for gastric damage.

## Introduction

The incidence of gastric injury has been increasing due to the widespread use of nonsteroidal anti-inflammatory drugs (NSAIDs). The mortality rate associated with gastric ulcer and other gastric injuries caused by NSAIDs is increasing by 0.22% per year (Wolfe et al. 1999). Therefore, it is crucial to alleviate gastric injuries induced by NSAIDs.

NSAIDs, such as indomethacin, are known to cause gastric injuries mainly because they block cyclooxygenase (COX)-1 and COX-2 (Sostres et al. 2013). COX-2-selective NSAIDs or multiple drug therapies may reduce these gastric adverse effects. However, selective COX-2 inhibition does not eliminate the risk of gastroduodenal ulcers and their complications (Lanas et al. 2007). Moreover, this approach offered little benefit to patients with a high susceptibility to NSAIDs-induced gastric injuries (Laine 2001; Malfertheiner et al. 2009; Harirforoosh et al. 2014), along with comparable or worse cardiovascular and renal toxicity (Abraham et al. 2007). The concomitant use of mucosal protective agents (e.g., misoprostol), H_2_ receptor antagonists (e.g., ranitidine), or proton pump inhibitors (e.g., omeprazole) could reduce the gastric or intestinal damage to a limited degree; however, further research is needed to prove their effectiveness (Harirforoosh et al. 2014).

The Chinese traditional herb *Alpinia officinarum* Hance (Zingiberoside) has been considered for treating NSAIDs-induced gastric injury due to its long history in the treatment of gastrointestinal diseases without obvious adverse effects (Ding 2015). *Alpinia officinarum* has been used as an antiemetic, stomachic, and analgesic in Asia for centuries (Kakegawa et al. 2014). We previously demonstrated that *A. officinarum* extract exhibits an antiulcer effect in an ethanol-induced gastric injury rat model (Wei et al. 2015). However, the mechanisms of *A. officinarum* extract in treating NSAIDs-induced gastric diseases remain unknown. Galangin is the most abundant flavonoid in the *A. officinarum* ethanol extract; it inhibits human neutrophil degranulation (Kanashiro et al. 2007), reduces mRNA levels of TNF-α, IL-1β, and IL-6 (Sivakumar and Anuradha 2011; Jung et al. 2014), and binds with COX-2 as indicated by a molecular docking study (Honmore et al. 2016). These results suggest that galangin may be a potent antiulcer compound. Thus, the purpose of this study was to assess the effects of *A. officinarum* extract and galangin on indomethacin-induced gastric injury, and to explore the underlying mechanisms.

## Material and methods

### Plant materials

*Alpinia officinarum* used in this study was harvested in August 2016 in Xuwen County, Guangdong Province of China. The plant was identified by Prof. Jianping Tian of Hainan Medical University. A voucher specimen (No. 20130716) was deposited at the School of Pharmacy of Hainan Medical University. The plants were air-dried at room temperature. The freshly dried rhizomes were manually shucked and mashed using a mashing machine (FW100, Taisite Instrument, Tianjin, China), and then sieved manually using a 60# mesh. The resulting fine powder and residue were mixed evenly.

### Preparation of plant extract

**Figure 1.**
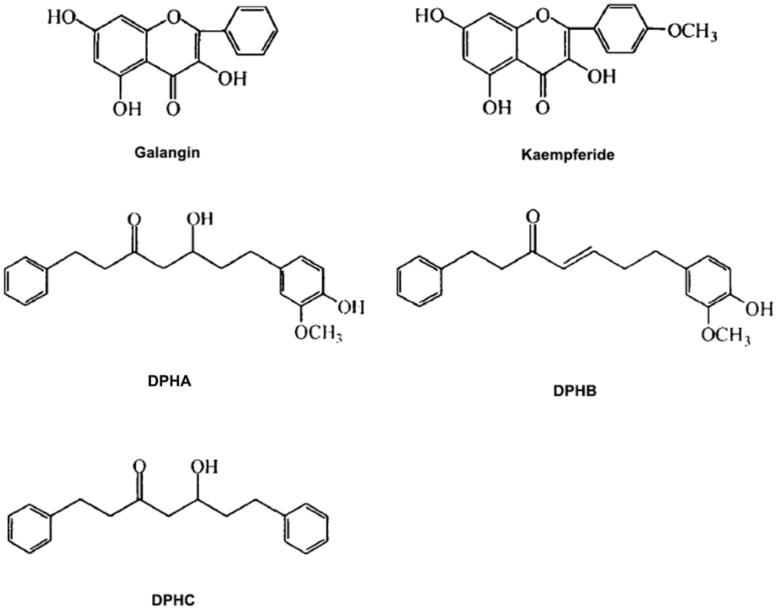
Chemical structures of five major constituents in *A. officinarum* ethanol extract. Galangin: 3,5,7-trihydroxyflavone; Kaempferide: 3,5,7-trihydroxy-4-methoxyflavone; DPHA: 5-hydroxy-7-(4-hydroxy-3-methoxyphenyl)-1-phenyl-3-heptanone; DPHB: 7-(4-hydroxy-3-methoxyphenyl)-1-phenyl-4-ene-3-heptanone; DPHC: 1,7-diphenyl-5-hydroxy-3-heptanone.

*Alpinia officinarum* ethanol extract was prepared using a protocol developed in our laboratory and was injected into the HPLC system for quantitative analysis (Cheng et al. 2015). An aliquot (1 kg) was weighed precisely and reflux extracted with 8-fold 80% ethanol for 1 h. The residue was extracted twice under the same conditions. The sampled ethanol extracts were combined and concentrated to 40% under reduced pressure. Then, the extract was purified with AB-8 macroporous resin by 80% ethanol. After the purification, polysaccharides, phenolic acids, and monosaccharides were removed, the proportion of flavonoids and diarylheptanoids in the extract was more than 50%. The final extract contained five major constituents, including galangin (11.8%), kaempferide (2.3%), 5-hydroxy-7-(4-hydroxy-3-methoxy phenyl)-1-phenyl-3-heptanone (cas: 68622-73-1, DPHA, 20.3%), 7-(4-hydroxy-3-methoxy phenyl)-1-phenyl-4-ene-3-heptanone (cas: 79559-60-7, DPHB, 5.1%), and 1,7-diphenyl-5-hydroxy-3-heptanone (cas: 24192-01-6, DPHC, 21.1%) (Figure 1).

Galangin was purchased from Chengdu Pufei De Biotech Co., Ltd. (Chengdu, China) due to the large requirement, the separation and extraction method was based on the protocol developed in our laboratory (Cheng et al. 2015).

### Animals

Six-week-old male and female Sprague-Dawley (SD) rats (Tianqing Biotechnology, Changsha, China) were housed (five animals per cage) under a 12 h light/dark cycle (07:00–19:00 photoperiod) at 25 °C and allowed *ad libitum* access to water and food. After 1 week of acclimatization, the 7-week-old rats, weighing 200–230 g, were used for experiments. All animal experiments were performed per International Guidelines for Care and Use of Laboratory Animals and were approved by the animal ethics committee of Hainan Medical University (reg. no. 201506017/HMU).

### Indomethacin-induced gastric injury rat model and treatment

Two groups of SD rats were randomly selected. One was a vehicle group (CON, *n* = 12, both sexes), and the other was a model group (MOD, *n* = 78, both sexes). The rats were fasted with water 24 h prior to experiments. Indomethacin (0.3 g/kg of body weight) was orally administered to rats in the MOD group. Seven hours after the administration, six rats (both sexes) from the model group were euthanized to confirm that gastric injury was established. The remaining model rats were divided into six groups. The groups were treated once daily with bismuth potassium citrate (0.08 g/kg, positive control drug, POS), galangin (0.2 g/kg, GAL), *A. officinarum* extract (0.03 g/kg, AOE-L; 0.09 g/kg, AOE-M; and 0.18 g/kg, AOE-H), or 1% (w/v) sodium carboxymethyl cellulose containing 2% glycerol (2.5 mL/kg of body weight, CON), respectively, for 6 days. Indomethacin (0.15 g/kg) was administered on Day 3 and Day 6 to maintain the gastric damage. During the entire experiment, rats in the CON group was administered an equivalent volume of vehicle solvent to confirm that the vehicle was not a potential confounder.

After the experiment, the rats were sacrificed and blood samples were withdrawn from the celiac artery. The blood samples were centrifuged at 3000 rpm for 15 min to obtain serum samples.

### Gross ulcer and histological indices

The stomachs were rapidly removed from the sacrificed rats, cut open along the greater curvature, and washed with ice-cold saline. The mucosal sides of the stomachs were photographed using a digital camera, and a part of the mucosa was immediately fixed with a 10% formalin solution. Gross damage to the gastric mucosa was assessed by two pathologists, who were blinded to the treatments, using a gross ulcer index (GUI, 1 point per 1 mm gastric injury). In addition, mucosal damage was graded based on histological injury index as follows: 0 (normal), 1 (mild), 2 (moderate), and 3 (severe).

### Western blot analysis of COX-1 and COX-2

Specimens of the mucosa were rapidly scraped from underlying gastric tissue layers using two glass slides, which were kept on ice. The mucosal tissues were weighed, minced by forceps, and homogenized in radio immunoprecipitation assay buffer (Thermo Scientific, Waltham, MA) containing 1% phenylmethanesulfonyl fluoride (Boster, Shanghai, China). The total protein concentration was determined using a BCA protein assay kit (Boster) and a BioTek microplate reader. A total of 50 µg of a protein lysate was resolved by 10% SDS–PAGE, and the proteins were subsequently transferred onto polyvinylidene difluoride membranes (EMD Millipore, Billerica, MA). The blots were blocked with 5% skim milk and probed overnight at 4 °C with the following primary antibodies: rabbit monoclonal anti-COX-1 (1:2000, Abcam, cat. no. ab109025; Cambridge, UK), rabbit polyclonal anti-COX-2 (1:2000, Abcam, cat. no. ab15191), and mouse monoclonal anti-β-actin (1:3000, Abcam, cat. no. ab8226). Then, the blots were washed with Tris-buffered saline containing Tween-20 and incubated with a goat anti-rabbit HRP-conjugated secondary antibody (1:4000, Abcam, cat. no. ab6721) or a goat anti-mouse HRP-conjugated secondary antibody (1:2000, Abcam, cat. no. ab6789) for 1.5 h at room temperature. Protein expression was quantified using a Tanon 4500 s fully automated chemiluminescence image analysis system (Tanon Technology Co., Ltd., Shanghai, China). The measurements were performed in duplicate for each experiment.

#### ELISA

Serum levels of TNF-α and VEGF were measured by ELISA kits (Abcam, cat. nos. ab100785 and ab100786, respectively). For cystathione-γ-lyase (CSE), ornithine decarboxylase (ODC), endothelin-converting enzyme-1(ECE-1), constitutive nitric oxide synthase (cNOS), and nitric oxide (NO) in gastric mucosa, appropriate kits from Abcam were used following the manufacturer’s instructions. Protein concentrations were measured using the BCA assay kit and a BioTek microplate reader.

### Statistical analysis

The results are presented as the mean ± standard deviation of the values obtained from 6 to 10 rats. Statistical analysis was performed by one-way analysis of variance (ANOVA) or Kruskal–Wallis *H*-test using the SPSS version 20 software (SPSS Inc., Chicago, IL). *p* values < 0.05 were considered significant in all cases.

## Results

### GUI and histological index

**Figure 2.**
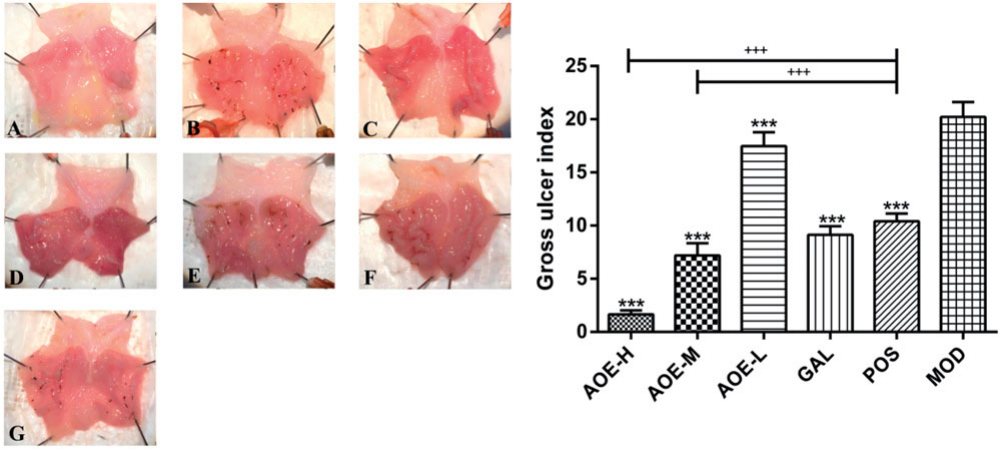
Gross findings of indomethacin-induced gastric damage in the rat gastric mucosa. Normal rat gastric mucosa (A) was damaged by the oral administration of indomethacin (B: 0.3 g/kg), and most of the damage was alleviated by treatment with *A. officinarum* extract (C: 0.18 g/kg; D: 0.09 g/kg; E: 0.03 g/kg) and galangin (F: 0.2 g/kg) after indomethacin administration. (G) Treatment with 0.08 g/kg bismuth potassium citrate. Healthy rat gastric mucosa in the control group (GUI of 0) are not shown. CON: vehicle control; AOE-H: 0.18 g/kg *A. officinarum* extract; AOE-M: 0.09 g/kg *A. officinarum* extract; AOE-L: 0.03 g/kg *A. officinarum* extract; GAL: 0.2 g/kg galangin; POS: 0.08 g/kg bismuth potassium citrate; MOD: 0.3 g/kg indomethacin. +++*p* < 0.001 compared with the POS group; ***p < 0.001 compared with the MOD group.

Mucosal shedding necrosis, edema, hyperemia, and inflammatory cell infiltration were observed in the gastric mucosa after indomethacin administration. *Alpinia officinarum* extract and galangin significantly decreased gastric mucosal injury to varying degrees (*p* <  0.001, Figure 2). *Alpinia officinarum* extract expressed a concentration-dependent therapeutic effect, and GUI decreased from 20.23 ± 1.38 to 1.66 ± 0.37 (AOE-H, 0.18 g/kg). A significant reduction in GUI was observed in the AOE-H and AOE-M treatment groups compared with that in the POS group (*p* <  0.001).

**Figure 3.**
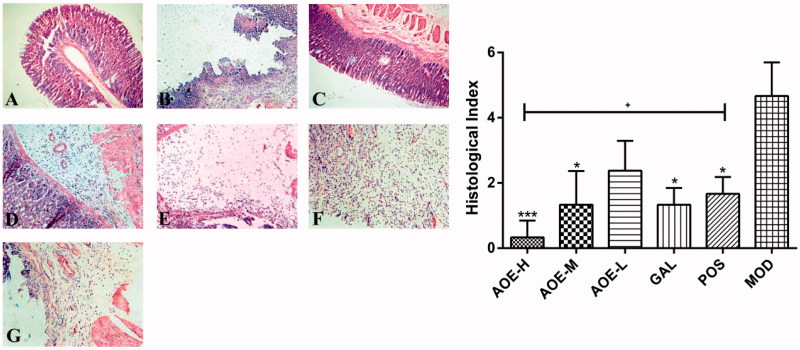
Histological findings (A–G) of the indomethacin-induced gastric damage in the rat gastric mucosa (magnification, 20×). Normal rat gastric mucosa (A) was damaged by the oral administration of indomethacin (B: 0.3 g/kg), and most of the damage was alleviated by treatment with *A. officinarum* extract (C: 0.18 g/kg; D: 0.09 g/kg; E: 0.03 g/kg) and galangin (F: 0.2 g/kg). (G) Treatment with 0.08 g/kg bismuth potassium citrate. Healthy rat gastric mucosa in the control group (histological index of 0) are not shown. CON: vehicle control; AOE-H: 0.18 g/kg *A. officinarum* extract; AOE-M: 0.09 g/kg *A. officinarum* extract; AOE-L: 0.03 g/kg *A. officinarum* extract; GAL: 0.2 g/kg galangin; POS: 0.08 g/kg bismuth potassium citrate; MOD: 0.3 g/kg indomethacin. +*p* < 0.05 compared with the POS group; *p < 0.05 and ***p < 0.001 compared with the MOD group.

Significantly reduced histological injury was observed in the AOE-H, AOE-M, GAL, and POS groups (*p* <  0.001, *p* <  0.05, *p* <  0.05, and *p* <  0.05, respectively, Figure 3). *Alpinia officinarum* extract also exhibited a concentration-dependent therapeutic effect, and the histological injury index was reduced from 4.67 ± 1.03 to 0.33 ± 0.51 after the treatment (AOE-H, 0.18 g/kg). When compared with the POS group, a significant reduction in this index was observed only in the AOE-H treatment group (*p* <  0.05). The two indexes indicated that *A. officinarum* extract and galangin ameliorated indomethacin-induced gastric injury, and the high-dose treatment with *A. officinarum* extract showed a better therapeutic effect than bismuth potassium citrate.

### COX-1 and COX-2 levels

**Figure 4.**
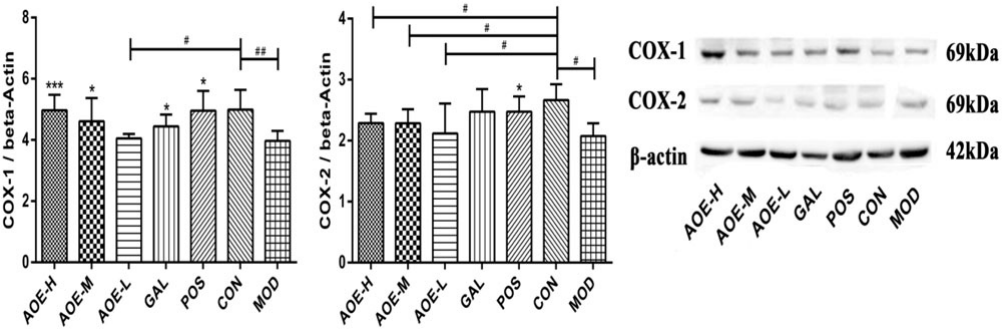
Western blot analysis of COX-1 and COX-2 in gastric mucosal tissues after 6 days of treatment. CON: vehicle control; AOE-H: 0.18 g/kg *A. officinarum* extract; AOE-M: 0.09 g/kg *A. officinarum* extract; AOE-L: 0.03 g/kg *A. officinarum* extract; GAL: 0.2 g/kg galangin; POS: 0.08 g/kg bismuth potassium citrate; MOD: 0.3 g/kg indomethacin. #*p* < 0.05 and ##*p* < 0.01 compared with the CON group; *p < 0.05 and ***p < 0.001 compared with the MOD group.

Gastric mucosal levels of COX-1 and COX-2 were both suppressed in indomethacin-treated rats (Figure 4). After 6 days of treatment, COX-1 levels in AOE-H, AOE-M, GAL, and POS groups were 1.25 (*p* <  0.001), 1.16 (*p* <  0.05), 1.12 (*p* <  0.05), and 1.25 (*p* <  0.05) times, respectively, higher than that in the MOD group. COX-1 levels in AOE-H, AOE-M, GAL and POS groups were not significantly different from that in the CON group (*p* >  0.05), indicating that COX-1 levels in these groups were restored to the normal level after the respective treatments. Among the five treatment groups, only COX-1 level in the AOE-L group was significantly lower than that in the POS group.

Only COX-2 levels in AOE-H and POS groups were 1.14 and 1.19 times, respectively, higher than that in the MOD group (*p* <  0.05). However, COX-2 levels in AOE-H, AOE-M, and AOE-L groups were statistically lower from that in the CON group (*p* <  0.05). When compared with the COX-2 level in the POS group, no statistical difference was found in the other treatment groups.

### Effects of the *A. Officinarum* extract and galangin on TNF-α, VEGF, ECE-1, cNOS, NO, CSE, and ODC levels

**Figure 5.**
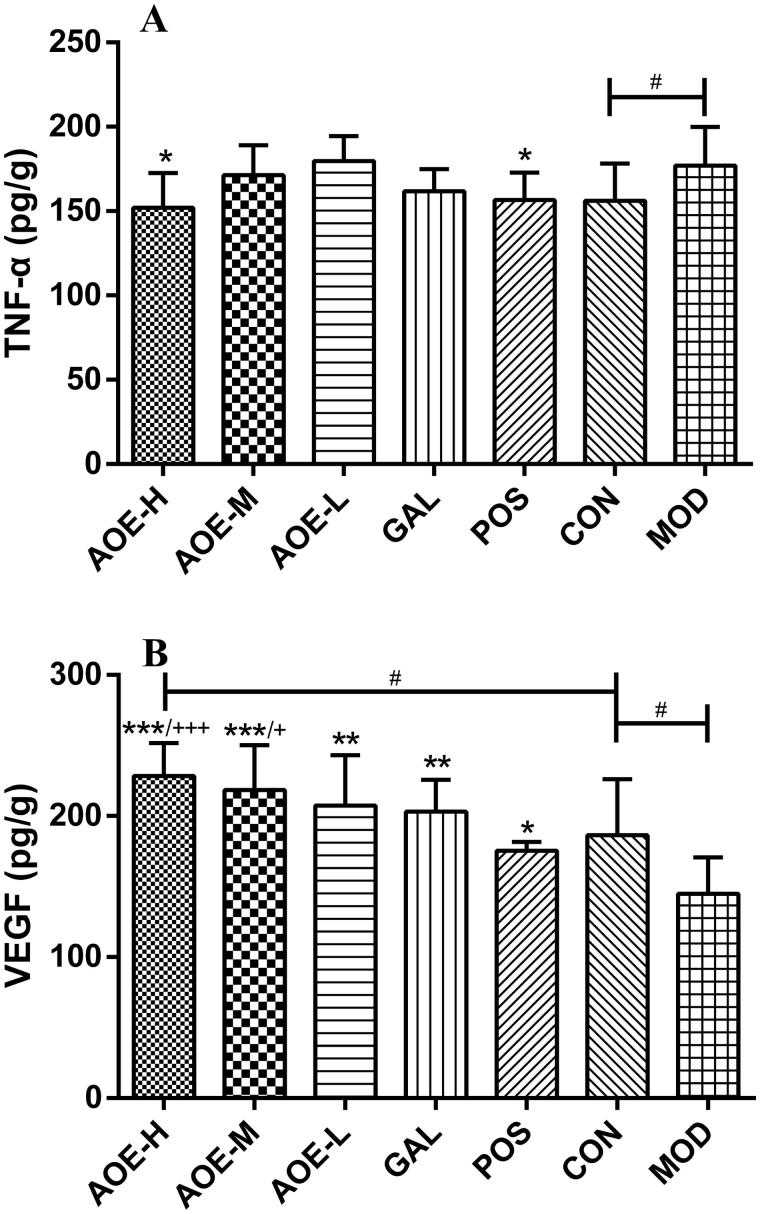
Effects of *A. officinarum* extract and galangin on TNF-α (A) and VEGF (B) levels in the serum. CON: vehicle control; AOE-H: 0.18 g/kg *A. officinarum* extract; AOE-M: 0.09 g/kg *A. officinarum* extract; AOE-L: 0.03 g/kg *A. officinarum* extract; GAL: 0.2 g/kg galangin; POS: 0.08 g/kg bismuth potassium citrate; MOD: 0.3 g/kg indomethacin. #*p* < 0.05 compared with the CON group; *p < 0.05, **p < 0.01, and ***p < 0.001 compared with the MOD group; +*p* < 0.05 and +++*p* < 0.001 compared with the POS group.

After 6 days of treatment, elevated serum levels of TNF-α observed after indomethacin administration were reduced in AOE-H and POS groups to 14.17 and 11.56%, respectively (*p* <  0.05). TNF-α levels in AOE-H, AOE-M, AOE-L, and GAL groups were not statistically different from that in the CON group (Figure 5(A)). Indomethacin-suppressed serum VEGF levels were significantly increased by *A. officinarum* extract and galangin treatments, and *A. officinarum* extract increased VEGF level in a dose-dependent manner; it was 1.57, 1.51, and 1.43 times higher in the AOE-H, AOE-M, and AOE-L groups, respectively, than that in the MOD group (Figure 5(B)). VEGF levels in AOE-H and AOE-M groups were significantly higher than that in the POS group (*p* <  0.001 and *p* <  0.05, respectively). Besides, the mean VEGF level in the AOE-H group was higher than that in the CON group (1.23 times, *p* <  0.05).

**Figure 6.**
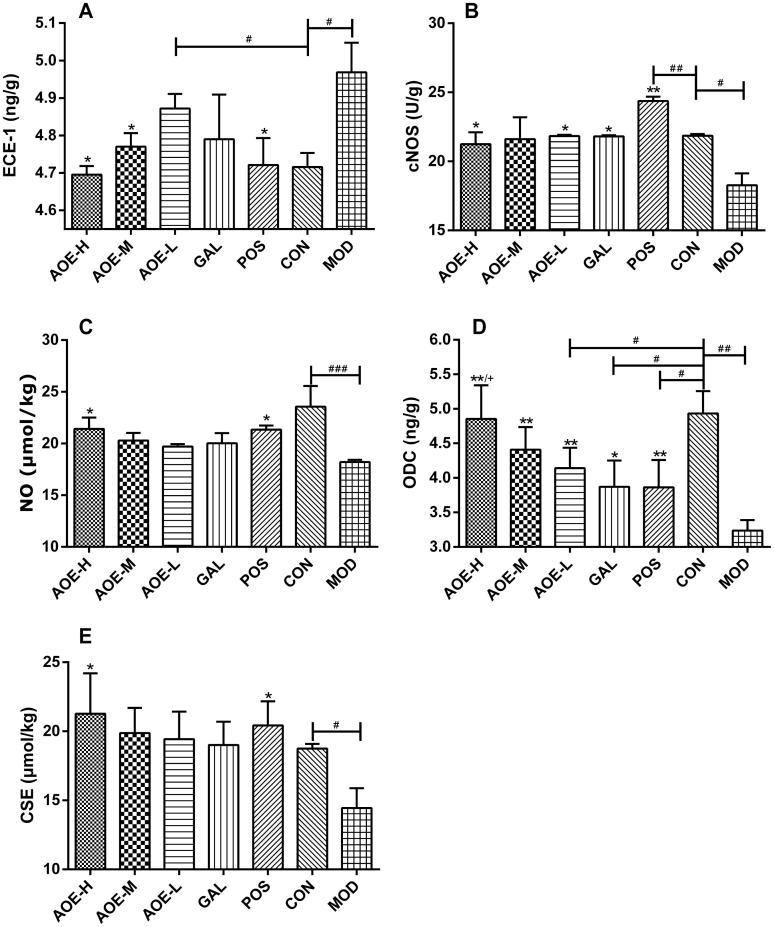
Effects of the *A. officinarum* extract and galangin on ECE-1 (A), cNOS (B), NO (C), ODC (D), and CSE (E) levels in the gastric mucosa. AOE-H: 0.18 g/kg *A. officinarum* extract; AOE-M: 0.09 g/kg *A. officinarum* extract; AOE-L: 0.03 g/kg *A. officinarum* extract; GAL: 0.2 g/kg galangin; POS: 0.08 g/kg bismuth potassium citrate; MOD: 0.3 g/kg indomethacin. #*p* < 0.05, ##*p* < 0.01, and ###*p* < 0.001 compared with the CON group; *p < 0.05 and **p < 0.01 compared with the MOD group; +*p* < 0.05 compared with the POS group.

In indomethacin-treated rats, gastric mucosal levels of ECE-1 were elevated (4.97 ± 0.08 ng/g), whereas cNOS and NO levels were significantly suppressed (18.27 ± 0.85 U/g and 18.20 ± 0.23 μmol/kg, respectively) (Figure 6(A–C)). AOE-H treatment prevented the elevation in ECE-1 and suppression in cNOS and NO levels caused by indomethacin (*p* <  0.05). In the CON group, gastric mucosal levels of ODC and CSE were 3.21 ± 0.15 ng/g and 18.74 ± 0.35 μmol/kg, respectively. Indomethacin administration significantly decreased ODC and CSE production (*p* <  0.01, Figure 6(D) and *p* <  0.05, Figure 6(E), respectively). After 6 days of treatment, ODC levels in the treatment groups remarkably increased, and ODC levels in AOE-H and AOE-M groups were restored to normal levels (*p* > 0.05 compared to the CON group). CSE levels also significantly increased in AOE-H and POS groups.

## Discussion

In this study, the effects and possible mechanisms of *A. officinarum* on indomethacin-induced gastric injury in rats were investigated. The GUI and histology results showed that *A. officinarum* extract and galangin alleviated indomethacin-induced gastric injury to varying degrees.

It is difficult to completely eliminate the adverse effects of NSAIDs because both their therapeutic roles and adverse reactions depend on the inhibition of COX enzymes. The inhibition of COX enzymes block the formation of endogenous prostaglandins (PGs) and related compounds, decreasing the gastric acid-mediated erosion of the mucosa, gastric mucosal blood flow, and carbonate synthesis and increasing susceptibility to mucosal injury and gastric ulceration (Takeuchi 2012; Sinha et al. 2013). Moreover, the acidic nature of indomethacin can directly kill epithelial cells and cause superficial injuries in the gastric mucosa (Tarnawski et al. 1988). Injured mucosa combined with COX inhibition can result in tissue ischemia, excessive bleeding, and acute inflammatory response (leukocyte adherence and inflammation) (Wallace and Granger 1996; Wallace 2008), which in return render the mucosa more susceptible to damage induced by indomethacin (Gana et al. 1987; Wallace et al. 2000). Increased inflammatory response results in an increased level of the proinflammatory mediator, TNF-α. This further leads to neutrophil adherence and the occlusion of gastric microvessels, thereby reducing gastric blood flow (Santucci et al. 1994; Sinha et al. 2013). Thus, VEGF becomes an essential factor in the ulcer-healing process owing to its angiogenesis function. However, VEGF release is mediated by PGs, which are produced via COX-2 pathway (Miura et al. 2004); therefore, the inhibition of COX-2 reduces VEGF production. The gastric mucosa then loses its resistance to damaging stimuli, resulting in the gradual formation of ulcers.

The present study confirms that NSAID-induced gastric damage is due to the inhibition of both COX-1 and COX-2 (Wallace et al. 2000; Gretzer et al. 2001; Tanaka et al. 2001). Therefore, the selective suppression of either isoform alone did not result in significant gastric damage in otherwise healthy animals. Our data showed that treatment with *A. officinarum* extract and galangin remarkably reverse the inhibition of COX-1 caused by indomethacin while having little influence on COX-2 levels, suggesting that *A. officinarum* and galangin may alleviate indomethacin-induced gastric injury by selectively increasing COX-1 levels while preserving the anti-inflammatory activity of indomethacin. Isolates of *A. officinarum*, such as galangal, kaempferide, DPHA, and DPHC, are potent COX-2 inhibitors according to molecular docking tests (Honmore et al. 2016); nevertheless, COX-2 levels in this experiment were not significantly depressed. One possible explanation may be that NSAIDs induce the acetylation of the COX-1 dimer, which alters its substrate specificity for the second dimer COX-2 (Yuan et al. 2009); therefore, the binding structures could have changed *in vivo*.

NSAIDs, such as indomethacin, also cause gastric damage by blocking the formation of H_2_S and NO, to weaken gastric mucosal integrity. H_2_S and NO are known to increase mucosal blood flow, stimulate mucus secretion, and inhibit neutrophil adherence (Martin et al. 2001). NO exerts a proangiogenic effect by stimulating VEGF production, whereas H_2_S could promote angiogenesis independent of VEGF (Malfertheiner et al. 2009). The inhibition of the mucosal synthesis of NO or H_2_S renders the stomach more susceptible to the damaging effects of NSAIDs and impairs the healing of preexisting damage (Wallace 2008; Musumba et al. 2009). Decreased H_2_S level is related to CSE inhibition in indomethacin-induced gastric injury. NSAIDs induce ulcerogenesis by increasing ECE-1 activity, thereby suppresses cNOS and endothelial NO (Musumba et al. 2009; Sinha et al. 2013). Besides, NO also mediates the production of H_2_S (Fiorucci et al. 2003). In this experiment, treatment with *A. officinarum* extract and bismuth potassium citrate (positive control) significantly prevented the induction of ECE-1, cNOS, NO and CSE levels caused by indomethacin, suggesting the possible mobilization and the involvement of NO and H_2_S formation pathways in the antiulcer effect of *A. officinarum*. Similar results have also been reported in drug-induced gastric ulcer models, where antiulcer effects were associated with significantly increased NO and H_2_S levels compared to control (Bayir et al. 2006; Kouitcheu et al. 2017). The administration of NO/H_2_S donors or NOS inhibitors increased the resistance of the gastric mucosa to injury induced by NSAIDs and other noxious substances and could accelerate the healing of ulcers in rodents (Fiorucci et al. 2005; Souza et al. 2004, 2008; Lowicka and Beltowski 2007). Besides, *A. officinarum* extract and galangin significantly increased VEGF levels suggesting that NO-stimulated VEGF could not only compensate for the decreased VEGF production by PGs, but also promote gastric blood flow. Taken together, these properties indicate important protective properties of NO and H_2_S in the gastric mucosa. Thus, *A. officinarum* may also exert its antiulcerogenic effect by increasing endogenous NO and H_2_S generation in the gastric mucosa.

In addition, ODC regulates the formation of polyamines, such as putrescine, spermidine, and spermine, which are essential for cell growth and proliferation, thereby recovering from gastric injury caused by NSAIDs (Lux et al. 1980; Musumba et al. 2009). Polyamine depletion may lead to the inhibition of both recovery from damage and epithelial cell proliferation, thereby delaying wound healing in the gastric mucosa (Saunders et al. 2008). After treatment, *A. officinarum* extract significantly replenished the depleted gastric ODC levels in a dose-dependent manner, and ODC level in the AOE-H group was similar to that in the CON group, indicating the involvement of ODC in the treatment effect of *A. officinarum*. Similar results were also observed after *Ganoderma lucidum* treatment in indomethacin- and acetic acid-induced gastric mucosal lesions (Gao et al. 2002). Studies showed that polyamines significantly and dose-dependently prevented the formation of gastric lesions induced by absolute ethanol (Brzozowski et al. 1991), and were implicated in the healing of stress lesions (Brzozowski et al. 1993). Exogenously administered spermine also significantly reduced gastriculcer index (by up to 90%) and normalized elevated acid secretion in rats treated with indomethacin (Musumba et al. 2009). Taken together, these properties indicate important protective properties of ODC and polyamines in the stomach. Thus, *A. officinarum* may also exert its antiulcerogenic effect by increasing the generation of endogenous polyamines through ODC level up-regulation.

Our results also indicate that the antiulcer activity of 0.2 g/kg galangin is weaker than that of 0.18 g/kg *A. officinarum* extract, suggesting that galangin may not be the primary antiulcer compound in *A. officinarum* ethanol extract. We hypothesize that DPHA and DPHC might be responsible for the antiulcer effect of the extract owing to their significant antioxidant and anti-inflammatory potential, and good binding with COX-2 (Honmore et al. 2016). Besides, PGs and polyamines as by-products of COX and ODC, respectively, should also be studied to further elucidate the mechanisms of action of *A. officinarum* in gastric protection.

Collectively, results of the present study indicate that *A. officinarum* and galangin treatment alleviate indomethacin-induced gastric damage in rats. The likely mechanisms include: (i) blocking the inhibitory effect of indomethacin on COX-1; (ii) increasing the generation of NO and H_2_S to increase mucosal blood flow (triggering the production of VEGF) and to inhibit leukocyte adherence; and (iii) reducing the inhibition of ODC expression.

## Conclusions

*Alpinia officinarum* and galangin alleviate indomethacin-induced gastric injury by regulating COX and non-COX signaling pathways. However, PGs and polyamines as by-products of COX and ODC, respectively, should also be studied to further elucidate the mechanisms of action of *A. officinarum* in gastric protection.
